# Plant Protein-Based Scaffolds for Cultivated Meat Production: A Review

**DOI:** 10.17113/ftb.64.03.26.9627

**Published:** 2026-09-15

**Authors:** Igor Slivac, Matija Maltarski, Kristina Radošević, Višnja Gaurina Srček

**Affiliations:** University of Zagreb Faculty of Food Technology and Biotechnology, Pierottijeva 6, 10000 Zagreb, Croatia

**Keywords:** cultivated meat, muscle cells, plant protein, scaffold fabrication, sustainability

## Abstract

This review summarises recent progress in the development of plant protein-based scaffolds for cultivated meat production, with a particular focus on protein sources derived from major agricultural crops and their processing into functional biomaterials. We discuss how protein extraction strategies, as well as emerging technologies, influence protein purity, functionality and suitability for scaffold fabrication. Current fabrication approaches, such as extrusion, freeze-drying, electrospinning, hydrogels and composite polymer systems, are examined in relation to their ability to generate three-dimensional, food-grade scaffolds with appropriate mechanical properties, porosity and cell compatibility. Key challenges and future research directions are also addressed, including the optimisation of plant protein-based scaffolds to support muscle cell adhesion, proliferation and differentiation, as well as the need for improved reproducibility and practical scalability. Overall, this contribution highlights the strong potential of plant protein-based materials to enable scalable cultivated meat production while reducing environmental impacts and reliance on animal-derived components.

## INTRODUCTION

Food availability and quality are fundamental to the sustainability of human society. Recent technological advances have enabled the production of food and agricultural products from cell cultures through cellular agriculture. Compared to conventional agriculture, cellular agriculture offers significant environmental benefits, including reduced greenhouse gas emissions, lower land and water use, decreased waste generation and improved food safety (*1*). A primary objective of cellular agriculture is the production of cultivated meat, also known as in vitro or cell-based meat, which aims to produce animal proteins in a sustainable, ethical and efficient manner using cell culture technologies (*2*). Cultivated meat production relies on four key components: animal-derived cells, growth media, three-dimensional (3D) scaffolds, and a scalable bioprocess (*3*).

Scaffolds are critical in cultivated meat production, as they provide a 3D framework that supports cell adhesion, proliferation, differentiation and tissue maturation, ultimately enabling a meat-like structure and texture (*4*). These scaffolds can be fabricated from animal-derived, plant-based or synthetic materials and are commonly composed of polysaccharides, proteins or polyesters (*5*). While animal-derived scaffolds such as gelatine, collagen, silk fibroin and decellularised extracellular matrices have been widely used, their application is limited by ethical concerns, batch variability, contamination risks, high costs, limited scalability and potential immunogenicity (*2*, *6*). These challenges highlight the need for alternative, non-animal scaffold materials.

Plant-protein-based scaffolds have emerged as promising alternatives due to their sustainability, cost-effectiveness, wide availability and favourable nutritional profiles (*7*, *8*). Properly structured plant proteins can mimic key characteristics of muscle tissue and support scalable cultivated meat production. However, plant proteins alone often lack sufficient mechanical stability, structural integrity and cell-adhesive properties, resulting in brittle or poorly interconnected scaffold networks (*9*-*11*). To address these limitations, food-grade polymers such as alginate, chitosan, gelatine or starch derivatives are frequently incorporated to enhance processability, mechanical strength, architectural stability and cell compatibility.

This review provides a comprehensive overview of recent advances in the use of plant-derived protein sources for scaffold fabrication in cultivated meat production. Although several reviews have addressed scaffold biomaterials for cultivated meat (*2*, *4*, *5*, *12*), to the best of our knowledge, none has focused exclusively on plant derived protein biomaterials. We summarise and critically compare recent developments involving proteins obtained from major agricultural crops, including corn, pea, soybean, wheat and others, with particular emphasis on their suitability for scaffold fabrication and their capacity to support the growth and functionality of animal-derived cells relevant to cultivated meat manufacturing. By examining fabrication techniques and material properties, this review highlights the potential of different plant proteins for the development of scalable, food-grade scaffolds tailored for cultivated meat applications. Given the rapidly growing interest in alternative protein materials, this work represents a timely contribution towards advancing more sustainable and efficient next-generation food technologies.

## REVIEW METHODOLOGY

We surveyed the literature using the Scopus, Web of Science, PubMed and Google Scholar databases, focusing on recent studies published mainly within the past five years. The search term “cultivated meat” was combined with the terms agro-industrial plants, scaffolds, microcarriers, legumes, cereals and proteins. Studies were selected for their relevance to the physicochemical, biological and processing properties of agro-industrial materials for cultivated meat production, with particular emphasis on peer-reviewed original research and review articles addressing the use of plant-derived protein sources in the development of cultivated meat scaffolds.

## PROTEIN SOURCES AND PROTEIN ISOLATION METHODS

Proteins are essential macronutrients that support growth and development and, beyond their nutritional role, their functional properties largely determine the texture, appearance and stability of food products. In this context, plant-based proteins represent a sustainable, cost-effective and health-oriented alternative to animal-derived proteins. They can be consumed directly or incorporated into foods to improve amino acid balance and nutritional quality. Growing efforts to meet increasing protein demand have therefore emphasised alternative protein sources, including their potential use as cell-supportive matrices in cultivated meat production, with cereals and legumes being the principal plant protein categories explored. By-products obtained after oil extraction from crops such as rapeseed, sunflower and pumpkin seeds are particularly promising due to their high protein content and potential for versatile valorisation of agro-industrial residues; however, to the best of our knowledge, they have not yet been explored for cultivated meat scaffold applications. Cereals like wheat, corn and sorghum generally contain lower protein amounts than legumes (5–15 %), but are widely utilised because of their favourable functional properties (*13*-*15*). Seed proteins in cereals and legumes are predominantly storage proteins, whose primary role is to supply nitrogen and amino acids to support seed germination and early plant growth. In cereals such as wheat and corn, storage proteins are mainly prolamins (e.g. gliadins, zein and kafirin) and glutelins, which together account for most of the total protein content and are characterised by high proline and glutamine amounts but limited essential amino acids, particularly lysine. Smaller fractions of albumins and globulins are also present in cereals and mainly contribute to metabolic and defence functions rather than storage. In contrast, legume seeds such as soybean, pea and peanut store proteins primarily as globulins, especially 7S vicilins and 11S legumins (*16*-*18*), which make up the majority of their protein content (15–30 %). These proteins provide a more balanced essential amino acid profile, although they may be relatively low in sulfur-containing amino acids. Legumes also contain minor amounts of albumins that function in enzymatic activity and seed defence. Overall, while cereal proteins are well adapted for energy storage and processing functionality, legume proteins offer higher protein content and improved nutritional quality, which underpins their growing relevance for food structuring applications (*19*, *20*).

Plant-based materials used for cultivated meat scaffold fabrication include protein isolates, defatted flour and protein-rich fractions obtained through protein extraction. Protein extraction from legume and cereal seeds is a critical step in producing plant-derived ingredients for food applications and emerging biotechnological uses, including cultivated meat scaffolds. These seeds contain abundant storage proteins embedded within complex matrices of starch, fibre and lipids, necessitating efficient extraction strategies. Conventional extraction approaches are broadly classified as dry or wet processes. Dry fractionation, based on milling and air classification, preserves native protein structures and minimises water and chemical use, but typically yields protein enrichments of 30-50 % (*21*). For lipid-rich seeds, defatting is often employed as a pre-processing step to improve protein accessibility and concentration (*22*, *23*). In contrast, wet extraction methods, commonly involving alkaline solubilisation followed by isoelectric precipitation, produce protein isolates of higher purity but require greater processing inputs and may cause partial protein denaturation.

In medium- to large-scale protein isolate production, where wastewater generation is a significant concern, protein solutions can be subjected to membrane-based concentration and separation processes such as ultrafiltration coupled with diafiltration. These techniques enable the efficient removal of co-extracted low-molecular-mass compounds while achieving high protein recovery and preserving the native structural and functional properties of proteins more effectively than conventional isoelectric precipitation (*24*, *25*). Emerging extraction strategies, including ultrasound-, enzyme-, microwave- and pressure-assisted techniques, have been developed to improve extraction efficiency and functionality (*26*, *27*). The primary outcome of these processes is a protein-rich fraction, typically obtained as a dry protein isolate with substantially enhanced protein content relative to the original biomass.

Overall, the transition from crude plant materials to protein isolates results in a substantial enrichment of protein content, with the final protein amount largely determined by the specific extraction and purification methods employed. While crude agro-industrial plant protein sources typically contain 15-30 % protein, isolation substantially increases the protein content, often resulting in a threefold enrichment. Accordingly, corn protein isolates generally contain 78–87 % protein (*28*-*30*), while pea and wheat protein isolates contain 68–90 % (*31*-*33*) and 55–72 % protein (*26*, *28*), respectively. Soybeans exhibit the highest crude protein content (32–43 %) among these sources, with protein amounts typically doubling during isolation (*34*, *35*). The residual non-protein components, such as fats, fibre and polyphenols that remain in the isolate may influence protein functionality, digestibility, and the structural and sensory performance of protein-based cultivated meat scaffolds (*36*).

## METHODS OF PROTEIN-BASED SCAFFOLD FABRICATION

Numerous techniques, with many variations, are used to produce scaffolds that support cell growth in cultivated meat research (*37*, *38*). Drawing from methods originally developed for tissue engineering and adapted for edible applications, these approaches aim to provide structural support, biocompatibility and nutritional value. The following sections present a brief overview of plant protein-based scaffold fabrication strategies explored for cultivated meat production. The references cited will primarily comprise recent review articles that summarise key advances and achievements in the relevant techniques.

### Solvent casting and crusting

A conventional strategy for fabricating cultured meat scaffolds involves casting a plant protein solution, often blended with a supporting polymer and a cross-linking agent (*39*). Solvent evaporation can yield polymer films of controlled thickness, whereas the production of bulk hydrogel structures typically requires additional pore-forming treatments to generate adequate porosity and interconnectivity. Porosity can be introduced either through particulate leaching, where food-grade porogens are incorporated into the cast protein matrix and subsequently removed to create interconnected, size-tunable pores, or through freeze-drying, in which the cast protein gel is frozen and lyophilised, generating a sponge-like structure *via* ice crystal templating. The principle of these methodologies lies in transforming plant proteins into a stable network around a temporary template: in particulate leaching, porogens such as sugar, salt or starch granules act as physical spacers that leave behind pores upon removal, while in freeze-drying, ice crystals formed during freezing serve as temporary templates that are removed through sublimation. Both approaches exploit the self-assembling and gel-forming properties of plant proteins to produce scaffolds with sufficient mechanical integrity and high porosity, providing a sponge-like environment that supports cell attachment, infiltration and nutrient transport.

Alternatively, protein-rich flour can be shaped into a solid, sheet-like structure using crusting. Crusting is a scaffold fabrication approach that avoids chemical additives and intensive machinery, relying on the self-assembly of soy biomolecules to form three-dimensional networks that support cell growth. To date, soy protein has been the primary material evaluated for crusting-based scaffold fabrication due to its high availability and favourable amino acid composition (*40*). In this process, ε-amino groups of amino acids in proteins react with reducing sugars through the Maillard reaction, enabling the formation of fibrous structures (*41*). During controlled heating and dehydration, a crust-like layer forms at the surface and gradually develops into a cohesive three-dimensional matrix with interconnected porosity. This naturally formed structure provides sufficient mechanical integrity and surface functionality to support cell attachment and proliferation without the need for external crosslinkers or complex processing steps.

### Fibre-forming methods

Production methods for micro- to nanoscale fibres from protein solutions enable precise tuning of scaffold morphology and mechanical properties to support cell adhesion, proliferation and tissue development (*42*). Among these, electrospraying, electrospinning and wet spinning are versatile fibre-forming techniques widely used to fabricate such fibrous scaffolds.

Electrospinning is a fabrication method that uses a high-voltage electric field to draw a polymer solution into fine fibres, creating nanofibrous scaffolds that closely mimic the fibrous structure of natural muscle tissue. In this process, a biomaterial solution (such as edible proteins or polysaccharides) is loaded into a syringe and ejected through a needle under high voltage, forming a charged jet. As the solvent evaporates, ultrafine fibres are deposited onto a collector, resulting in a porous, mesh-like scaffold. These scaffolds provide a large surface area, support cell adhesion, growth and alignment, and can be tailored for texture and mechanical properties suitable for cultured meat applications. Edible, biocompatible materials are preferred to ensure safety and functionality in the final meat product (*43*). Beyond the production of continuous fibres by electrospinning, electrospraying is a related electrohydrodynamic technique that enables the generation of particulate structures for scaffold design. A high-voltage electric field is used to produce fine droplets from a polymer or hydrogel solution, resulting in microspheres or microbeads that can act as scaffolds for cell culture. In this method, a biomaterial solution (such as alginate-gelatine) is fed through a needle and subjected to an electric field, which causes the liquid to break into uniformly sized, charged droplets. These droplets are gathered in a coagulation bath (which typically contains calcium ions for crosslinking), where they harden into microspheres. The final microbeads can encapsulate cells or be arranged into larger 3D structures, offering a supportive, edible and biocompatible environment for the growth of muscle cells and tissue development in cultured meat applications. Electrospraying enables precise control over particle size and cell distribution and is regarded as a gentle, cytocompatibile method appropriate for scalable scaffold production. Owing to its precise control over particle size and gentle processing conditions, electrospraying is widely regarded as a cytocompatible and scalable method for microcarrier fabrication (*44*). Zein, and to a much lesser extent gliadin, dominate electrospinning research among plant proteins, due to their hydrophobic nature and excellent fibre-forming ability, while other proteins are usually applied in composite systems to achieve sufficient viscosity, conductivity and mechanical integrity for scaffold applications.

Wet spinning with plant proteins involves dissolving or dispersing the proteins in a suitable solvent, often with pH adjustment and blending with biopolymers to improve spinnability (*45*). The protein solution is extruded through a spinneret into a coagulation bath containing a nonsolvent or crosslinking ions, where the proteins precipitate and form continuous fibres. Solvent–nonsolvent exchange induces fibre solidification and partial protein denaturation, stabilising the structure. The fibres are then collected, washed to remove residual solvent or salts and optionally stretched to enhance alignment and mechanical strength. Finally, the fibres may be dried or kept hydrated depending on their intended application, such as edible scaffolds for cultivated meat.

### 3D-printing and extrusion

3D printing has emerged as a promising approach for fabricating edible scaffolds from plant proteins for cultivated meat applications, as it enables precise control over scaffold geometry, porosity and mechanical properties. The most common technique is extrusion-based printing, which is widely used due to its compatibility with viscous plant protein inks. In these methods, plant protein-based inks derived from sources such as soy, pea, wheat or oilseed proteins are tailored to achieve suitable rheological properties for stable deposition and shape fidelity. However, plant proteins alone often show limited printability due to insufficient viscosity, poor shear-thinning behaviour or weak structural integrity after deposition. To overcome these limitations, plant proteins are frequently blended with other biopolymers, such as alginate, gelatine, cellulose derivatives or starch, which improve rheological performance, extrusion stability and shape retention (*46*). Supporting polymers, plasticisers or cross-linking agents are often incorporated to enhance printability and post-printing stability. Layer-by-layer fabrication enables the formation of interconnected pore networks that support cell attachment, nutrient diffusion and tissue maturation. Following printing, post-processing steps such as thermal treatment, enzymatic or ionic cross-linking and controlled drying are applied to fix the scaffold structure and improve mechanical strength. Overall, 3D printing of plant protein-based edible scaffolds offers high design flexibility and tunability, making it a versatile platform for cultivated meat research and development (*47*).

Alternatively, plant proteins from soy, pea and certain oilseeds can be subjected to a process involving heat, pressure and shear to form fibre-structured products. During extrusion, proteins denature and realign, creating a fibrous network that mimics the texture of muscle tissue, commonly referred to as textured vegetable protein (TVP) (*48*, *49*). In low-moisture extrusion, typically below 30 % water content, the heated protein-rich mass expands upon exiting the extruder due to a sudden pressure drop, forming a sponge-like TVP. This material is then dried to ensure shelf stability and can be rehydrated prior to use. In contrast, high-moisture extrusion operates at water contents above 50 % and uses cooling to prevent expansion, yielding dense, anisotropic, meat-like structures with aligned fibres that can be consumed as meat analogues (*50*). In the context of cultivated meat, low-moisture TVP can be cut into defined shapes, rehydrated and further functionalised to serve as an edible, porous scaffold that supports cell attachment, nutrient diffusion and three-dimensional tissue development.

### Microcarriers

Microcarriers are small, solid or porous particles widely used in animal cell technology to provide a high surface area for the attachment and expansion of adherent cells in bioreactor systems. In protein-based microcarrier manufacturing, emulsification is commonly used to produce spherical particles by dispersing an aqueous protein phase into an immiscible continuous phase, followed by gelation or solidification to stabilise particle morphology (*51*). Plant protein-based microcarriers can also be produced by emulsification or by adapting techniques, such as spray drying and extrusion, often combined with crosslinking or blending with food-grade polymers (e.g. gelatine) to tailor mechanical stability and surface characteristics, thereby supporting scalable cultivated meat production (*52*).

## PLANT PROTEIN SOURCES FOR CULTIVATED MEAT SCAFFOLD FABRICATION

### Corn proteins

Zein, the major prolamin storage protein derived from corn, has attracted considerable interest as a sustainable and cost-effective material for scaffold fabrication. Biologically, zein is a heterogeneous mixture of proteins with variable molecular masses, solubilities and charges. Its high content of non-polar and uncharged amino acids, including glutamine, alanine, leucine and proline, contributes to its pronounced water insolubility (*14*). Zein has been extensively investigated in both nutritional and biomedical applications, particularly in the development of innovative food products, bioactive membranes, wound healing and drug delivery systems (*53*). Owing to its natural origin, excellent biocompatibility and biodegradability, zein has also been explored as a scaffold material for cultivated meat production, including in films, electrospun sheets and hydrogels. Its inherent hydrophobicity makes zein suitable for the fabrication of sheet-like scaffolds *via* electrospinning, a technique that has been extensively investigated in tissue engineering for over two decades. Biocompatibility studies using mesenchymal stem cells have demonstrated effective differentiation into adipogenic, chondrogenic, and osteogenic lineages, supporting the concept of structured tissue formation relevant to cultivated meat products (*54*, *55*).

The functional properties of zein, including its film-forming ability and self-assembly, enable the fabrication of scaffolds with tunable mechanical strength, porosity and surface topography, all of which are favourable for mammalian cell adhesion and growth, including muscle cells. However, the intrinsic hydrophobicity of zein can limit cell attachment and nutrient diffusion. These limitations can be addressed through fibre crosslinking or by combining zein with hydrophilic polymers, thereby enhancing both biocompatibility and mechanical performance (*56*) (Table 1 (*57*-*60*)). In this context, zein-alginate fibres were produced *via* a wet-spinning method, in which a 1 % hydrophilic sodium alginate solution was injected into a coagulation bath containing zein and CaCl2 (*57*). The zein coating significantly improved tensile strength, elastic modulus, and strain tolerance compared with uncoated alginate fibres. Thirty percent zein was identified as optimal for providing a protective and mechanically robust coating. The resulting zein-alginate fibres successfully supported adhesion, proliferation and differentiation of bovine satellite cells (BSCs), adipocytes and endothelial cells, corresponding to the primary cellular components of muscle, fat and vascular cell types, respectively. This multi-lineage support demonstrates the potential of zein-alginate fibres to mimic the complex cellular architecture of meat in cultivated meat applications. Similarly, electrospun short-stranded zein fibres (SSFs) were incorporated into arginine-glycine-aspartic acid (RGD)-functionalised alginate hydrogels to enhance the cellular environment for BSC growth (*58*). The addition of 0.1 % zein SSFs to 1.8 % RGD-functionalised alginate hydrogels improved hydrogel degradation behaviour and strengthened interactions between primary BSCs and the gel matrix, as demonstrated by enhanced cell spreading, alignment and F-actin expression. In contrast, increasing the fibre content to 0.5 % reduced these benefits, likely due to fibre aggregation and consequent changes in hydrogel structure. This approach of integrating short-stranded zein fibres into alginate hydrogels offers a promising strategy for promoting 3D tissue formation in cultivated meat applications by improving scaffold structural stability and extending culture duration.

**Table 1:** The main properties of zein-based scaffolds for cultivated meat production

Scaffold fabrication	Supporting polymer (and cross-linker)	Key properties	Cell type	Reference
Wet-spinning	Sodium alginate, CaCl_2_	Zein 30 %, tensile stress 0.68 MPa, elastic modulus 3.04 MPa, biocompatible	C2C12, BSCs, BASCs, endothelial cells	(*57*)
Electrospinning	RGD-alginate	Zein 28 %, fibre diameter 600 nm; fibre size distribution 0.202 µm, biocompatible	BSCs	(*58*)
3D printing	Hordein, secalin	Pore size 400 µm, fibre height 7-10 µm, porosity up to 88 %, highly ordered, water-stable, degradable, biocompatible	C2C12,PSCs	(*59*)
Filming	Fucoidan, TGase	Enhanced hydrophilicity, good cell adhesion rate, minimal degradation, structural integrity, biocompatible	C2C12	(*60*)

BSC=bovine satellite cells, BASC=bovine adipose stem cells, RGD=arginine-glycine-aspartic acid, PSC=porcine satellite cells, TGase=transglutaminase

To develop innovative prolamin-based bioinks with improved printability and structural performance, zein was combined with higher molecular mass prolamins, including hordein from barley and secalin from rye (*59*). Printing parameters were optimised to produce edible zein-hordein and zein-secalin scaffolds with well-defined structures and favourable mechanical properties. The resulting scaffolds showed good water stability, controlled in vitro degradability, and a fibrous structure without requiring additional crosslinking. The cytocompatibility of these prolamin scaffolds was evaluated using both C2C12 and porcine satellite cells (PSCs). The scaffolds supported cell adhesion, proliferation, differentiation and fusion into tissue-like structures, as demonstrated by increased expression of adhesion-related genes and the presence of myogenic proteins. These effects were attributed to the combination of the hydrophilic properties of the scaffolds and the bioactive influence of scaffold-associated peptides on cellular behaviour. Furthermore, PSCs cultured on prolamin scaffolds formed layered tissue structures resembling meat slices, demonstrating both the functional potential and the commercial relevance of these scaffolds for cultivated meat production. Consistent with ongoing efforts to enhance the biofunctionality of zein-based scaffolds, zein was recently combined with fucoidan to create composite scaffolds that support myoblast attachment and differentiation (*60*). Fucoidan is a natural sulfated polysaccharide obtained from brown algae and has attracted considerable interest for applications in the food industry and biomedicine due to its bioactivity and biocompatibility (*61*). The resulting zein-fucoidan scaffolds exhibited a stable 3D structure with improved hydrophilicity and functional properties, leading to enhanced C2C12 cell adhesion, with attachment efficiency increasing from 44 to 81 %. In addition to improved cell attachment, C2C12 cells cultured on zein-fucoidan scaffolds demonstrated increased proliferation and enhanced myogenic differentiation, as evidenced by greater myotube length and fusion index. Mechanistic studies revealed that fucoidan promotes cell proliferation through activation of the extracellular signal-regulated kinase (ERK) pathway, mediated by regulation of key phosphorylation processes within the JNK and p38 MAPK signalling pathways. Overall, these findings provide mechanistic insight into the role of fucoidan in regulating myoblast behaviour and support its integration with plant-based proteins, such as zein, as a promising strategy for scaffold design in cultivated meat production.

### Pea protein

Owing to its edibility, low allergenicity, cost-effectiveness and biodegradability, pea protein, derived from the seeds of Pisum sativum, has emerged as a promising functional and sustainable scaffold material for cultivated meat production. Pea seeds contain approx. 20–25 % protein, primarily composed of globulins (60–70 %) and albumins (15–20 %). Pea protein exhibits a balanced essential amino acid profile and is particularly rich in lysine, contributing to its favourable nutritional value. Currently, pea protein is widely used in the food industry as an emulsifier and gelling agent, as well as a substitute for soy- or animal-derived proteins in various functional food products (*17*). Beyond its nutritional and functional attributes, pea protein isolate (PPI) contains diverse functional groups that support animal cell adhesion, making it an attractive candidate for scaffold fabrication in cultivated meat applications (Table 2 (*7*, *62*-*65*)).

**Table 2:** The main properties of pea protein-based scaffolds for cultivated meat production

Scaffold fabrication	Supporting polymer (and cross-linker)	Key properties	Cell type	Reference
Cooling	Polysaccharides (agarose, gellan and xanthan locust bean gum)	Stable gels up to 1 % soy and pea protein, increased nutritional value, good stability in medium for 7 days, cytocompatible, cell encapsulation	C2C12	(*62*)
3D printing	RGD-alginate, CaCl_2_	Increased scaffold stability and stiffness, biocompatible	BSCs	(*7*)
	Pectin+gum Arabic, CaCl_2_	Viscoelastic solid behaviour, shear-thinning and microstructure recovery, good biocompatibility	C2C12	(*63*)
Freeze-drying	Alginate, CaCl_2_	Large scaffold size, porosity ~80 % (at pH=2), high liquid absorption ~400 %, modulus ~8.5 kPa, high nutritional value (pea protein 13.4 %), low allergenicity	BSCs	(*64*)
	Tannic acid, glycerol	Porous structures, good textural properties (hardness 3.84 N, gumminess 1.15–2.74 and chewiness 0.64–1.24) and digestibility	C2C12	(*65*)

BSC=bovine satellite cells, RGD=arginine-glycine-aspartic acid

In addition to soy protein isolate (SPI), the effects of incorporating PPI into polysaccharide-based hydrogels have also been investigated. Wollschlaeger *et al*. (*62*) examined the effects of up to 1 % PPI on the stability of hydrogels formulated from agarose, gellan or a mixture of xanthan and locust bean gum (1, 1 and 0.5 %, respectively). Hydrogels containing 1 % PPI exhibited enhanced nutritional value and favourable mechanical properties, while higher PPI amounts led to unstable gel formation. Encapsulation studies with C2C12 myoblasts demonstrated high cell viability, with the majority of cells remaining viable during three days of culture.

In a related study, PPI was combined with RGD-modified alginate (1:1 m/V) to fabricate scaffolds using 3D printing for the promotion of BSC growth and differentiation (*7*). The results indicated that PPI enhanced scaffold stability and stiffness, thereby supporting efficient cell attachment and maturation. Moreover, the authors developed enriched PPI-based bioinks with well-defined geometries that successfully supported cell recovery and maturation within 3D-printed cellular constructs. In the formulation of bioinks, pea protein acts primarily as a rheology modifier and structural binder, improving the printability and shape fidelity of the bioink during extrusion-based 3D printing. In a study where gelatine-based microcarriers were confluent with fish muscle satellite cells and adipose-derived stem cells, pea protein contributed to the formation of a cohesive, tissue-like construct through crosslinking with microbial transglutaminase (TGase), while also enhancing the nutritional value of the final cultured fish fillet (*66*). In a more recent study, Kim *et al*. (*63*) developed a hydrogel composed of PPI and pectin and assessed its cytocompatibility using C2C12 myoblasts. The scaffolds demonstrated favourable rheological properties, including viscoelastic solid behaviour, shear thinning characteristics and microstructure recovery, which are essential for 3D printing. Moreover, the PPI-pectin scaffold showed good cytocompatibility and supported C2C12 proliferation, without significant differences compared to the cells cultured on standard tissue culture plates.

Pea protein tends to form highly viscous solutions at elevated amounts, and its solubility is strongly pH-dependent, with minimum solubility observed in the pH range of 4–6 (*17*). To overcome these limitations while enhancing the protein content and nutritional value of scaffolds, David *et al*. (*64*) developed an edible scaffold using a freeze-drying approach that combined 15 % PPI at pH=2 and 7 with 2 % alginate. The resulting scaffolds were evaluated for BSC proliferation and differentiation. Although both formulations exhibited low cell proliferation and metabolic activity (<5 %), they supported myogenic differentiation, with the pH=7 scaffold showing higher myosin heavy chain expression than the pH=2 scaffold. Another example of a PPI-based scaffold with elevated protein content and natural polyphenol crosslinking was reported by Kim *et al*. (*65*). In that study, scaffolds containing two PPI mass fractions (12 and 15 %) were fabricated using a freeze-drying method and crosslinked with tannic acid at concentrations of 0, 0.38 or 0.75 mg/mL. Chemical and mechanical analyses revealed that tannic acid significantly influenced the gelation behaviour of PPI, as well as the resulting scaffold microstructure and surface characteristics. Incorporation of tannic acid led to improved mechanical strength, while the textural properties of the scaffold were comparable to those of conventional meat and meat analogues. These findings are consistent with a previous report demonstrating that tannic acid enhances scaffold mechanical stability, with its antioxidant properties potentially contributing to improved shelf life and oxidative stability of the final meat product (*67*). Furthermore, in vitro digestibility assessment indicated that the amino acid release profile during simulated gastric digestion was comparable to that of PPI gels, suggesting preserved nutritional value. Cytocompatibility assessments showed that the scaffolds supported C2C12 cell attachment and proliferation over a 5-day culture period. However, no specific cell orientation was observed during cell growth, indicating potential limitations related to scaffold topography or the absence of biological features necessary for cellular alignment.

### Soy proteins

Soy protein is derived from soybeans after oil extraction and is widely used as an alternative to animal-derived proteins. It consists of a heterogeneous mixture of proteins with molecular masses ranging from 140 to 300 kDa, exhibiting diverse physicochemical properties (*16*). Most soy proteins are classified as storage proteins, with more than 90 % comprising globulins that vary in both size and abundance. In addition, soy protein contains arginine-glycine-aspartic acid (RGD) sequences and is rich in glycine and proline, amino acids that are generally essential for cell adhesion and proliferation (*68*). Owing to its favourable biocompatibility, non-toxicity, versatility and sustainability, soy protein has been extensively investigated as a biomaterial for medical applications, including drug delivery, wound healing and tissue engineering (*69*, *70*). Furthermore, due to its adaptability, edibility and advantageous physicochemical properties, such as gelation behaviour and thermal crosslinking capacity, soy protein has emerged as a promising plant-based material for scaffold fabrication and application in cultivated meat technology. The key properties of soy protein-based scaffolds relevant to cultivated meat production that have been investigated in recent studies are summarised in Table 3 (*40*, *62*, *71*-*77*).

**Table 3:** The main properties of soy protein-based scaffolds used for cultivated meat production

Scaffold fabrication	Supporting polymer (and cross-linker)	Key properties	Cell type	Reference
Extrusion	-	Edible, porous, supports 3D tissue, meat-like texture	BSCs BSMc, endothelial cells	(*71*)
Crusting	Vegetable glycerol	Inexpensive, minimal machine processing requirement, spontaneous folding, variable pore size, biocompatible	C2C12	(*40*)
Freeze-drying	TGase, CaCl_2_	Pore size 50–250 μm, porosity 72–83 %, compressive moduli 3.8–4.2 kPa, stabile for 30 days in PBS, no need for cell adhesion agents	C2C12	(*72*)
	Chitosan	Pore size 50–250 μm, porosity 71–78 %, compressive modulus 4.8–15.3 kPa, stable for 3 months in PBS, biocompatible	C2C12	(*73*)
	Soy dietary fibre, TGase, CaCl_2_	Porosity ~73 %, liquid absorption ~309 %, enhanced stability	NIH 3T3	(*74*)
	Carrageenan and sodium alginate, CaCl_2_/KCl	Porosity 59–77 %, swelling ratio over 700 %, scalable, low-cost, simple, energy-efficient process	C2C12	(*75*)
	Agarose and agar	Stable gels at 5 % soy and pea protein, stable structure, high water-holding capacity, good compressive strength	PADSCs	(*76*)
Cooling	Agarose, gellan and xanthan locust bean gum	Stable gels up to 1 % soy and pea protein, increased nutritional value, good stability in medium for 7 days, cytocompatible, cell encapsulation	C2C12	(*62*)
Microcarriers (filming+mixing+sieving)	TGase, glycerol	Homogeneous and dense structure, large surface area for cell attachment	C2C12,BSCs	(*77*)

BSC=bovine satellite cells, BSM=bovine smooth muscle cells, PBS=phosphate buffered saline, TGase=transglutaminase, PADSC=porcine adipose-derived stem cells

One form of soy protein used for scaffold fabrication is textured soy protein (TSP), an extruded product widely used as a meat analogue in food applications. TSP has an inherently porous microstructure and can be readily processed into various shapes and sizes, making it a suitable substrate for cell attachment and growth. Accordingly, TSP has been investigated as an edible scaffold material for the proliferation and differentiation of bovine satellite cells (BSCs) in culture media supplemented with myogenic growth factors (*71*). Compared with BSC monoculture, co-culturing BSCs with bovine smooth muscle and endothelial cells resulted in significantly enhanced myogenesis, as indicated by increased myogenin expression and increased production of extracellular matrix (ECM) proteins. Mechanical characterisation of TSP cell scaffolds revealed properties comparable to those of native bovine muscle tissue. Furthermore, sensory evaluation by volunteers indicated a meat-like flavour and texture, underscoring the sensory acceptability of the resulting cultivated meat product.

Beyond the use of TSP, the intrinsic self-assembling properties of soy protein have also been exploited to fabricate edible scaffolds for cultivated meat. In this context, another research group used crusting, a non-chemical and low-energy processing technique, to produce soy protein-based scaffolds (*40*). The authors developed scaffolds composed of pure soy homogenate (Y2R) and soy homogenate supplemented with 1 % mass fraction of vegetable glycerol (Y2G). The resulting scaffolds were characterised in terms of pore size, water absorption capacity and compressive properties, with Y2R scaffolds exhibiting superior structural and mechanical performance. Both scaffold formulations were further evaluated for myogenic C2C12 cell proliferation and differentiation, demonstrating good biocompatibility and supporting myotube formation. However, during the differentiation phase, the scaffolds were susceptible to degradation, indicating the need for further optimisation of their mechanical properties. Nonetheless, that study demonstrated that soy protein, as well as potentially other plant-derived proteins and carbohydrates, can serve as substrates for scaffold fabrication using inexpensive techniques such as crusting, thereby offering a promising strategy to reduce the overall production costs of cultivated meat.

In addition to fibrous and self-assembled soy protein-based scaffolds, gel-based scaffolds have attracted considerable attention in cultivated meat production due to their structural similarity to the ECM, which offers high water retention, increased porosity and specific molecules favourable for cell adhesion (*78*). These scaffolds are typically derived from natural polymers, including plant-derived proteins, and are often combined with functional, food-grade additives such as dietary fibre, polyphenols and enzymes (e.g. transglutaminase) to enhance their mechanical properties and biocompatibility (*67*). Given the intrinsically limited mechanical strength and water resistance of soy proteins, functionalisation or blending with other materials is frequently used. Accordingly, the mechanical properties of soy protein-based gels can be improved by incorporating additional biopolymers, such as polysaccharides or synthetic polymers, or through chemical or enzymatic crosslinking strategies (*4*, *15*). These approaches enable the fabrication of scaffolds with stiffness and elasticity comparable to those of native muscle tissue, which are critical parameters for effective muscle function and maturation. Among available crosslinkers, TGase is commonly employed to modify soy proteins through the formation of ε-(γ-glutamyl) lysine crosslinks, thereby reducing scaffold degradation over time and improving structural stability (*69*). Consistent with this approach, Wei *et al*. (*72*) developed porous amyloid fibril-based scaffolds composed of soy β-conglycinin (7S), glycinin (11S) and SPI. The scaffolds were double crosslinked using microbial TGase and CaCl2, followed by temperature-controlled water vapour annealing to generate 3D hydrogel structures. This fabrication approach yielded stable, highly porous scaffolds with pore sizes ranging from 50 to 250 μm, porosities of 72–83 % and compressive moduli between 3.8 and 4.2 kPa, depending on the specific soy protein used. The resulting scaffolds supported proliferation and differentiation of mouse C2C12 myoblasts, showing high metabolic activity as assessed by the Presto Blue viability assay. Notably, the use of food-grade enzymatic and ionic crosslinking agents emphasises the potential of abundant, low-cost soy protein amyloid fibrils for cultivated meat applications without the need for additional cell-adhering molecules. In a similar approach, soy β-conglycinin amyloid fibrils were crosslinked with the natural polysaccharide chitosan to fabricate scaffolds and evaluate their mechanical and biological performance (*73*). Chitosan interacts effectively with negatively charged protein solutions through electrostatic interactions and hydrogen bonding, thereby enhancing scaffold mechanical strength and promoting cell adhesion (*79*). Depending on the physicochemical properties of chitosan, including its degree of deacetylation and molecular mass, the resulting soy amyloid fibril-based scaffolds exhibited tunable pore sizes ranging from 50 to 250 µm, porosities between 71 and 78 %, and compressive moduli ranging from 4.8 to 15.3 kPa. The scaffolds maintained their structural integrity during storage in refrigerated phosphate-buffered saline (PBS) for up to three months. In addition to their stability, the scaffolds showed good bioactivity by supporting the proliferation and differentiation of C2C12 myoblasts.

Soy protein isolate (SPI), a highly refined form of soy protein containing more than 90 % protein, exhibits favourable gelation behaviour and thermal crosslinking capacity, enabling the fabrication of gel-based scaffolds. Fang *et al*. (*74*) successfully developed double-crosslinked hydrogel scaffolds composed of SPI and soy dietary fibre (SDF), a natural polysaccharide derived from soybean, using TGase and/or calcium ions as crosslinking agents. Compared with individually crosslinked scaffolds, the double-crosslinked scaffolds exhibited enhanced mechanical strength and water absorption capacity (up to 309.45 %), while maintaining a highly porous structure (72.66 %). The biocompatibility of SPI-SDF scaffolds was evaluated using mouse embryonic fibroblast NIH 3T3 cells through assessment of cell proliferation and cytotoxicity. The results showed that these scaffolds supported cell adhesion and promoted the expression of genes associated with cell migration and adhesion, indicating their suitability for cultivated meat applications. More recently, a traditional Ito-Kanten (thread agar) manufacturing approach, based on two-step directional freezing and ionic crosslinking, was adapted to fabricate porous hydrogel scaffolds composed of SPI, carrageenan (CA), and sodium alginate (SA) (*75*). In this process, SPI, carrageenan and sodium alginate were dissolved in water, thermally treated, homogenised and subjected to directional freezing in liquid nitrogen. The frozen gels were subsequently immersed in Ca2+ and K+ solutions for low-temperature ionic crosslinking, followed by a second freezing step and lyophilisation, yielding anisotropic cryogel scaffolds. The resulting SPI/CA/SA scaffolds exhibited high porosity (59–77 %), swelling ratios exceeding 700 % and favourable mechanical properties. Moreover, the scaffolds effectively supported C2C12 myoblast attachment, proliferation and differentiation. Overall, these findings demonstrate the suitability and scalability of SPI-based hydrogel fabrication strategies that rely on food-grade components and do not require expensive equipment or toxic reagents, underscoring their strong potential for application in cultivated meat production.

To develop food-grade hydrogels with enhanced nutritional value and tunable functionality, combinations of multiple plant proteins have been studied, particularly soy protein and pea protein. De Berardinis *et al*. (*80*) investigated the co-gelation behaviour of soy protein and pea protein, focusing on the effects of protein amount, pH and ionic strength on scaffold formation. The results were organised into a comprehensive gelation map, providing a practical basis for the production of soy protein/pea protein hydrogels for food-related applications, including cultivated meat production. This systematic approach highlights the advantages of multi-protein systems in enabling precise control over gelation behaviour and final scaffold properties. In a related study, Wollschlaeger *et al*. (*62*) examined the influence of SPI and PPI on the stability of polysaccharide-based hydrogels composed of agarose, gellan and xanthan-locust gum, materials known for forming stable hydrogels with adjustable mechanical properties. The incorporation of SPI (up to 1 %) resulted in hydrogels with stable physical and rheological properties. Cytotoxicity assays of scaffold leachates and encapsulated C2C12 myoblasts revealed no adverse effects, confirming their biocompatibility. These findings underscore the compatibility of soy protein/pea protein systems with established polysaccharide networks, further expanding their applicability in cultivated meat scaffold fabrication. Similarly, Kim *et al*. (*76*) employed SPI and PPI in hydrogel fabrication and evaluated their gelation behaviour, physical properties, cytotoxicity and capacity to support cell adhesion using porcine adipose-derived mesenchymal stem cells (ADSCs). The results demonstrated superior cell attachment and proliferation on SPI-based agarose scaffolds compared with PPI-based scaffolds. This enhanced bioactivity was attributed to the presence of lunasin peptides in SPI, which contain RGD motifs known to promote cell adhesion. Overall, these studies highlight the advantages of dual plant protein-based scaffolds, in which soy protein contributes bioactive motifs that enhance cell adhesion, while pea protein offers complementary nutritional and functional attributes. Such synergistic combinations enable the development of nutritionally enriched, mechanically tunable and biologically active food-grade hydrogels, reinforcing their promise as scalable and versatile scaffold materials for cultivated meat production.

Recent studies have highlighted the importance of developing edible, food-grade, animal-free microcarriers (MCs) for large-scale cultivated meat production (*52*). MCs are small, 3D particles that provide a surface for anchorage-dependent cells to attach and proliferate in suspension culture, thereby enabling scalable production of cultivated meat. In this context, SPI-based MCs have been developed and their ability to support the attachment, proliferation and differentiation of mouse and bovine myoblasts has been evaluated (*77*). MCs were prepared by dispersing SPI in water, followed by heating and crosslinking with TGase and glycerol to form stable SPI films. The films were then processed in a Thermomix with 70 % ethanol, homogenised and passed through a stack of sieves (600 and 400 μm mesh) or gelled to form stable microparticles. The resulting SPI microcarriers showed sufficient mechanical stability for use in stirred bioreactors and successfully supported bovine myoblast attachment, proliferation and differentiation, even under serum-free culture conditions. Functionally, SPI microcarriers were comparable to commercially available collagen-coated dextran microcarriers, with a 24-fold increase in bovine myoblast concentrations observed over 8 days of culture. These results demonstrate that SPI-based MCs are not only scalable and mechanically robust but also fully edible and food-grade, making them a highly promising platform for large-scale cultivated meat production.

### Wheat proteins

Wheat gluten typically comprises 75–85 % protein, with minor amounts of carbohydrates and lipids. Its primary protein components are glutenins, which form high-molecular-mass aggregates stabilised by disulfide bonds, and gliadins, which exist predominantly as monomeric proteins. Glutenins are particularly rich in glutamine and proline, but contain relatively low amount of charged amino acids, a property that contributes to their poor solubility and strong intermolecular interactions (*13*).

Due to their biocompatibility, food-grade safety, nutritional value and cost-effectiveness, glutenins have attracted attention as promising plant-based scaffold materials for cultivated meat production. However, the highly crosslinked structure of glutenins presents significant challenges for solubilisation and further processing (Table 4 (*81*-*84*)). To overcome this limitation, glutenin has been processed into 3D porous sponges and aligned fibrous scaffolds using a freeze-drying approach (*81*). By adjusting the glutenin amount, the resulting scaffolds exhibited tunable pore sizes ranging from 50 to 250 μm and compressive moduli between 0.5 and 1.9 kPa. Importantly, the scaffolds maintained structural integrity in PBS for at least six months, demonstrating their suitability for long-term cell culture applications. Moreover, the glutenin-based scaffolds supported the proliferation and differentiation of C2C12 and BSc myoblasts without additional adhesion proteins or surface-coating molecules. Wu *et al*. (*82*) developed glutenin-chitosan 3D porous scaffolds with adjustable stiffness and microstructural features for cultivated meat applications. The mechanical properties and pore architecture of the glutenin-chitosan scaffolds were systematically modulated by varying the glutenin/chitosan ratio, resulting in tunable pore sizes and compressive moduli. Structural characterisation revealed a well-defined and interconnected porous structure, while spectroscopic analyses demonstrated increases in α-helix and β-sheet content, strengthened hydrogen-bonding interactions, and enhanced disulfide cross-linking with increasing glutenin amounts. These molecular and structural changes jointly contributed to improved scaffold stiffness and stability. Biocompatibility evaluation showed that the glutenin-chitosan scaffolds supported the growth and myogenic differentiation of PSCs, as confirmed by cell viability assays and immunofluorescence staining. Overall, glutenin-chitosan 3D scaffolds exhibit tunable mechanical and structural properties, promote muscle cell proliferation and differentiation, and closely mimic the textural characteristics of native meat, offering promising opportunities for cultivated meat production.

**Table 4:** The main characteristics of glutenin-based scaffolds used for cultivated meat production

Scaffold fabrication	Supporting polymer (and cross-linker)	Key properties	Cell type	Reference
Freeze-drying	_­_-	Pore size 50–250 μm, modulus 0.5–1.9 kPa, stable for 6+ months, food-grade, no need for cross-linkers	C2C12, BSCs	(*81*)
	Chitosan	Pore size 18–67 μm, modulus 16–60 kPa (tunable), the increscent content of α-helix and β-sheet, organised porous structure	PSCs	(*82*)
Extrusion	Corn starch	Highly aligned fibrous structure, elastic modulus close to native muscle, water-holding capacity	C2C12	(*83*)
Filming	-	Tunable thickness, preserved β-sheet conformation, high thermal stability, long-term stability (for 28+ days)	C2C12, 3T3-L1	(*84*)

BSC=bovine satellite cells, PSC=porcine satellite cells

Ikuse *et al*. (*83*) investigated the use of extruded plant protein blends as two-dimensional scaffolds to support muscle cell growth for cultivated meat applications. Five protein formulations were evaluated, including soy protein from two different sources as well as wheat-, mung bean- and faba bean-derived proteins. All scaffolds exhibited fibrous, anisotropic structures characteristic of extrusion processing, although fibre alignment varied depending on the protein source. By day 14 of culture, all scaffolds supported the attachment and proliferation of C2C12 myoblast. Notably, the wheat-based scaffold demonstrated superior myoblast growth, which was attributed to its highly aligned fibrous structure and an elastic modulus comparable to that of native skeletal muscle tissue. In a related study, Yao *et al*. (*84*) fabricated glutenin-based films with and without surface patterns to investigate their capacity to regulate C2C12 myoblast behaviour. Both patterned and unpatterned films effectively supported the alignment and organisation of C2C12 myoblasts without the need for surface modifications using cell-adherent peptides (e.g. RGD) or ECM proteins. Furthermore, the authors examined the dynamics of cell−ECM interactions, which play an important role in determining the functional and organoleptic properties of the final cultivated meat product. Their results revealed increased collagen deposition by both C2C12 myoblasts and 3T3-L1 preadipocytes after 14 and 28 days of culture. These findings provide valuable insight into the interplay among scaffold materials, cellular composition and culture duration, and highlight their combined effects on the nutritional value and textural properties of cultivated meat.

### Other plant-based proteins

Attempts have also been made to develop scaffolds for cultivated meat production from plant proteins obtained from less commonly used sources (Table 5 (*85*-*90*)). For example, chickpea proteins (CP) are attracting attention as sustainable, nutritive and functional substitutes for animal proteins. Their favourable amino acid profile, hypoallergenic properties and versatile functional properties make them promising candidates for both cultivated meat scaffold and plant-based meat analogue production. Food-grade microcarriers made from chickpea protein hydrolysates *via* electrospraying, and further modified with trypsin, were used to demonstrate their potential to support the growth of C2C12 cells, PSCs, chicken satellite cells, and 3T3-L1 cells (*85*). The microcarriers facilitated cell differentiation into muscle or fat cells under optimal cultivation conditions. Moreover, trypsin improved the cytocompatibility of the chickpea protein hydrolysates by exposing lysine and arginine residues to interact with cell surface receptors and facilitate cell proliferation through the activation of signalling pathways. Using electrospraying and electrospinning techniques, chickpea protein was used to fabricate edible microcarriers (CP-MCs) and fibrous scaffolds (CP-FSs) to create minced and thick-cut cultivated meat products (*86*). Spherical CP-MCs with an average diameter of 577 μm were obtained, while CP-FSs formed structures composed of isotropic or aligned elastic fibres with an average width of 4.5 μm. Chickpea protein was not cytotoxic to cells and improved the adhesion and proliferation of mesenchymal stem cells and skeletal muscle cells. The scaffolds retained the key proteins from chickpea protein, along with their molecular structures and thermal behaviour. Additionally, both scaffold types were able to support cell growth, allowing complete coverage of CP-MCs within 10 days of culture and an 8-fold increase in cell number on CP-FSs. In accordance with the above studies, chickpea protein and its hydrolysate showed potential for cultivated meat production as cost-effective and scalable biomaterials.

**Table 5:** Less frequently used protein sources for cultivated meat scaffold research

Protein	Scaffold fabrication		Key properties	Cell type	Reference
Chickpea	Coating		Improved cytocompatibility by trypsin treatment, biocompatible	C2C12, PSCs, chicken satellite cells, 3T3-L1	(*85*)
	Electrosprayed microcarriers, electrospun fibres		Preserved protein structure and composition, stability, good cell adhesion and proliferation	BMSCs, C2C12	(*86*)
Kafirin	Porous sponge freeze-drying		Interconnected pores, improved cell infiltration, good cell adhesion and differentiation, improved sensory properties	SMC, ADSC	(*87*)
Peanut		Wire drawing	Fibrous structure, improved cell adhesion and differentiation properties	ADSC, SMC	(*88*-*90*)

PSC=porcine satellite cells, BMSC=bovine mesenchymal stem cells, SMC=skeletal muscle cells, ADSC=adipose-derived stem cells

Another plant-based protein used as a promising biomaterial for cultivated meat production is kafirin, a hydrophobic prolamin protein isolated from sorghum. Kafirin’s high hydrophobicity, strong disulfide cross-linking and self-assembling capacity enable the formation of 3D porous scaffolds with high mechanical strength and interconnected pores, which are essential for muscle and fat cell proliferation and differentiation in cultivated meat production. These scaffolds can be fabricated from sorghum grain and dried distillers’ grain with solubles (DDGS) left over from bioethanol production (*15*). Recent research (*87*) has shown that kafirin can be effectively used to fabricate 3D porous sponge-like scaffolds using a simple template-leaching technique for the cultivation of PSCs and ADSCs. The scaffolds contain interconnected pores with high porosity (84 %) and good mechanical properties. ADSCs and PSCs adhered, proliferated and differentiated within the protein scaffolds, as confirmed by morphological observations and expression of regulatory factors. Subsequently, a hybrid cultivated meat was developed by cultivating ADSCs on kafirin scaffolds for 12 days. Anthocyanins, naturally present in red sorghum, enhanced the hybrid cultivated meat with meat-like colour and antioxidative properties. Additionally, hybrid cultivated meat prototype exhibited improved sensory, nutritional and textural characteristics, emphasising the feasibility of using sorghum prolamin in cultivated meat production.

The growing range of plant protein sources for cultivated meat scaffold development now also includes peanuts. Scaffolds fabricated from fibres drawn from a viscous peanut protein solution exhibited appropriately fibrous and porous morphology that mimics the structure of muscle tissue. These so-called wire-drawing protein scaffolds have been successfully used to support the attachment and proliferation of PSCs (*88*) as well as the mass culture of porcine ADSC, resulting in a final product with improved texture and a volatile composition closely resembling real meat (*89*). The same research group reported that peanut-based scaffolds exhibited superior adhesion of porcine smooth muscle cells (SMC) compared with scaffolds derived from soybean and wheat proteins, supporting the production of both ECM and muscle proteins. However, cell proliferation and differentiation declined with increasing passage number (*90*).

## CHALLENGES AND FUTURE PROSPECTS

In cultivated meat technology, scaffolds provide the structural framework for cell attachment, proliferation and organisation, thereby defining product texture independently of protein content and other nutritional variables. The fabrication method is decisive for both performance and commercial feasibility. As summarised in Fig. 1, hydrogel moulding combined with physical or chemical crosslinking and modest post-processing (thermal setting or freeze-drying) remains the dominant route. It is of low complexity, food-grade compatible and well suited to water-soluble proteins such as soy and pea. More hydrophobic proteins, including zein and peanut protein and, to a lesser extent, chickpea protein, are better matched to fibre-forming methods such as electrospinning, which offers finer control of architecture and surface features relevant to myogenic alignment. Zein combines high solution viscosity with shear-thinning behaviour and is therefore a strong candidate for three-dimensional printed constructs; blending with alginate, cellulose derivatives or pectin can improve print fidelity and shape retention, while gelatine or recombinant adhesive motifs may still be needed to enhance attachment. Extrusion of soy, wheat and chickpea proteins is gaining attention for its scalability and capacity to generate aligned, mechanically robust structures with reduced reliance on supporting polymers. Microcarriers produced by emulsification or electrospraying remain a promising but underexplored option for non-structured products. Protein selection cannot rest on processability alone. Amino-acid profile, digestibility and nutritive value must be considered if the scaffold is to contribute to the edible product, and scale-up must balance cost against resource efficiency and life-cycle impact. Limited cell adhesion remains the principal barrier for scaffolds made solely of plant proteins. Collagen, gelatine and fibrinogen are highly cell-supportive, yet they conflict with the animal-welfare rationale of cultivated meat; recombinant proteins and fungal biomass therefore offer more consistent animal-free alternatives (*91*, *92*). A further constraint is the grassy, beany or nutty off-notes typical of soy and pea, which arise from lipid oxidation products and endogenous volatiles and can persist through fabrication of cultivated meat products. Mitigation includes highly purified isolates, low-oxidation extraction, less flavour-active proteins such as zein, flavour-masking ingredients or fat-containing cell biomass, and post-processing flavour optimisation (*93*). Pea protein is currently among the strongest candidates for industrial scaffolds, combining availability, cost-effectiveness, established food-grade supply and favourable gelation, water retention and rheology. Mechanical and cell-interactive limitations can be addressed by blending with other food-grade polymers or bioactive components, yielding composite hydrogels, freeze-dried porous matrices or printable bioinks. Longer-term sustainability will require diversification beyond conventional protein-rich crops. This need may be addressed by valorising proteins recovered from leafy green plants and algae, which grow rapidly, yield high biomass throughout the year and are frequently discarded as agricultural residues or left unused. Finally, integrating proteome analysis with economical processing can accelerate identification of proteins that combine structural stability, digestibility and intrinsic cell-adhesive motifs, reducing the need for extensive chemical modification and improving translational readiness.

**Fig. 1:**
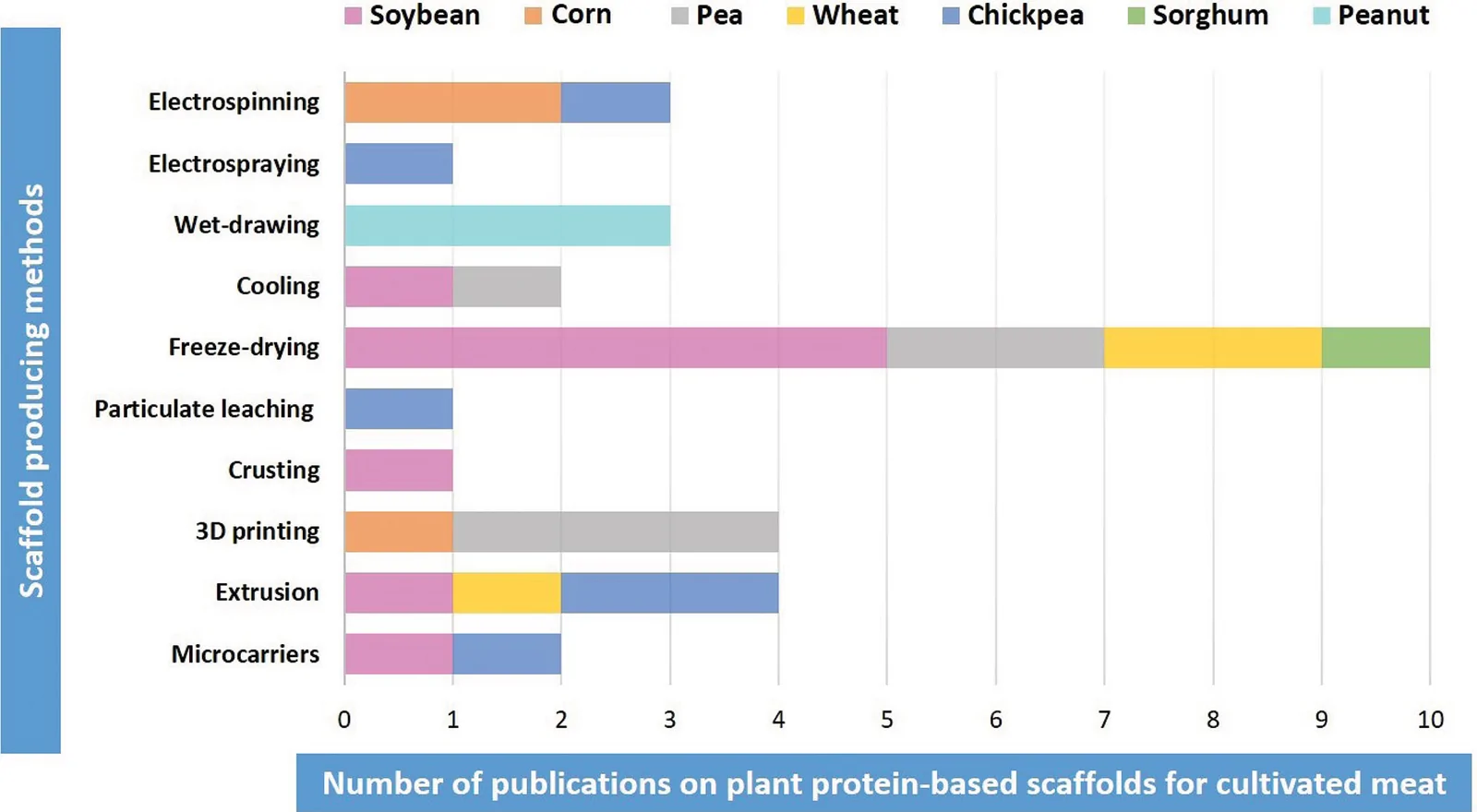
Current trends in the development of plant protein-based scaffolds for cultivated meat: a literature survey (2020–2026)

## CONCLUSIONS

This review has examined the current state of plant protein-based scaffolds for cultivated meat, with particular emphasis on how protein source, physicochemical properties and fabrication route jointly determine scaffold architecture, cell-supportive performance and industrial viability. Plant proteins offer a food-grade, potentially scalable and animal-free alternative to conventional extracellular matrix materials, yet they are not interchangeable. Water-soluble globular proteins such as soy and pea are readily processed into hydrogels and porous matrices, whereas more hydrophobic proteins such as zein are better exploited through fibre-forming and printing technologies. Across this landscape, fabrication method is as consequential as composition: hydrogel moulding remains the most accessible and food-compatible platform, while electrospinning, extrusion and emerging microcarrier approaches provide complementary routes to aligned, mechanically robust or high-surface-area constructs. Two persistent bottlenecks constrain translation. First, many unmodified plant proteins display limited cell adhesion relative to collagenous matrices, which continues to motivate composite design, recombinant motif display and fungal or microbial co-materials. Second, characteristic plant-derived off-flavours, together with incomplete control of texture and nutritional contribution, must be resolved if scaffolds are to be retained in the edible product rather than treated as sacrificial templates. Pea protein currently offers the most balanced combination of availability, cost, processing versatility and consumer familiarity for near-term structured applications, provided it is formulated as part of a composite system. Looking ahead, progress will depend on coupling materials science with proteomics, life-cycle assessment and sensory engineering, and on expanding the feedstock base to include underutilised leafy and residual biomass. Taken together, plant protein scaffolds are not a single solution but a tunable platform: when fabrication strategy, protein functionality and nutritional design are aligned, they can support cultivated meat products that are structurally convincing, nutritionally relevant and consistent with the sustainability and animal welfare objectives of the field.
